# A crosstalk between phosphorylation and ubiquitination of BNIP3 regulates mitophagy under hypoxia

**DOI:** 10.1080/27694127.2023.2197637

**Published:** 2023-04-04

**Authors:** Yun-Ling He, Jian Li, Yan Cao, Hai-Tao Wu, Li-Ying Wu

**Affiliations:** a Department of Neurobiology, Beijing Institute of Basic Medical Sciences, Beijing, China; b Beijing Institute of Microbiology and Epidemiology, Beijing, China; c State Key Laboratory of Proteomics, Beijing Proteome Research Center, Beijing Institute of Radiation Medicine, Beijing, China; d Chinese Institute for Brain Research, Beijing, China

**Keywords:** BNIP3, hypoxia, mitophagy, phosphorylation, ubiquitination

## Abstract

BNIP3 (BCL2/adenovirus e1B 19 kDa protein interacting protein 3) is a mitochondrial outer membrane protein that is sensitive to hypoxia and mediates mitophagy, a process important for mitochondrial quality control and to maintain energetic and redox homeostasis under hypoxia. It has been reported that up-regulation of BNIP3, which acts as mitophagy receptor, promotes mitophagy. In our recent study, we found that the post-translational modification of BNIP3 is crucial to induce mitophagy, and that a crosstalk between phosphorylation/dephosphorylation and ubiquitination acts as a switch to control BNIP3-mediated mitophagy under hypoxia. We demonstrated that the phosphorylation of BNIP3 at S60 and T66 by MAPK8/9 (mitogen-activated protein kinase 8/9) under hypoxia blocks the degradation of BNIP3 via the ubiquitin-proteasome pathway and enhances its interaction with MAP1LC3 (microtubule associated protein 1 light chain 3), thereby promoting mitophagy. In contrast, dephosphorylation of BNIP3 by members of the PP1/2A (protein phosphatase PP1 and PP2A) phosphatase subfamily under hypoxia accelerates degradation of BNIP3 via the ubiquitin-proteasome pathway, thereby suppressing mitophagy. Altogether, these findings provide knowledge necessary to devise intervention strategies for hypoxia-related diseases and/or hypoxia-related developmental processes.

**Abbreviations:** BCL2: BCL2 apoptosis regulator; BCL2L1: BCL2 like 1; BECN1: beclin 1, autophagy related; BH3: BCL2 homology 3; BNIP3: BCL2/adenovirus e1B 19 kDa protein interacting protein 3; LIR: MAP1LC3-interacting region; MAP1LC3: microtubule associated protein 1 light chain 3; MAPK8: mitogen-activated protein kinase 8; MAPK9: mitogen-activated protein kinase 9; PEST: rich in amino acids P, E, S, T, and D; PP1: protein phosphatase 1; PP2A: protein phosphatase 2A; PEST: rich in amino acids P, E, S, T, and D;

Mitophagy is recognized as a protective metabolic mechanism through which dysfunctional mitochondria are cleared to maintain the mitochondrial quality control. Thus, mitophagy plays an important role in many pathophysiological processes, including inflammation, cancer, aging, stroke and neurodegenerative disorders. Mitophagy is regulated by a spectrum of mitophagy receptors under different conditions. BNIP3 is one of them and it mostly operates under hypoxia conditions.

BNIP3 is mainly located at the outer mitochondrial membrane and belongs to the BCL2 (BCL2 apoptosis regulator) protein family. BNIP3 has several structurally defined regions, including the LIR (MAP1LC3-interacting region), the PEST (rich in amino acids P, E, S, T, and D), the BH3 (BCL2 homology 3) and the TM (transmembrane) domains. Numerous studies have revealed a key role of BNIP3 in regulating mitophagy under hypoxia. In particular, the up-regulation of BNIP3 levels by HIF-1 promotes its interaction with both MAP1LC3 and BCL2/BCL2L1 (BCL2 like 1), leading to the release of BECN1 (beclin 1, autophagy related) from BCL2/BCL2L1, which cooperatively promote the initiation and progression of mitophagy. In addition to its expression level, the post-translational modification of BNIP3 also plays a critical role in the induction of mitophagy. Previous studies have demonstrated that the phosphorylation at S17 and S24, which is adjacent to the LIR motif, mediates the association of BNIP3 with MAP1LC3 and regulates mitophagy. More recently, it has been reported that the phosphorylation at T181 of the mouse BINP3, which is homologous to T188 in the human protein and adjacent to the TM, changes the subcellular localization of BNIP3 and this redistribution may be involved in regulation of mitophagy. However, little was known about the role and mechanism of post-translational modification of BNIP3 under hypoxia. Our recent study reveals that the phosphorylation of rat BNIP3 at S60 and T66, which are homologous to S66 and T72 in the human protein and localized within the PEST domain, blocks its degradation via the ubiquitin-proteasome pathway, which in turn induces mitophagy. These observations show that the crosstalk between phosphorylation and ubiquitination of BNIP3 regulates mitophagy under hypoxia [1].

Using an oxygen-sensitive PC12 cell line, we found that hypoxia conditions do not continuously induce mitophagy. In particular, we observed that mitophagy is activated at early stage of hypoxia and inactivated after prolonged hypoxia. At the same time, we observed that BNIP3 displayed different migration pattern on SDS-PAGE gels under different hypoxia durations. While the apparent molecular weight of BNIP3 was 30 kDa in the early stages of hypoxia, it changed to 21.5 kDa after prolonged hypoxia. We showed that phosphorylation and dephosphorylation of BNIP3 is what leads to different migration profile on SDS-PAGE gels by treatments with phosphatase inhibitor and Lambda phosphatase, respectively. Furthermore, we demonstrated that BNIP3 phosphorylation under hypoxia correlated with an induction of mitophagy. Exploiting mass spectrometry database and site-mutation screening, we found that the phosphorylation of BNIP3 at S60 and T66 is regulated by hypoxia. Expression of a BNIP3 variant with phospho-mimicking mutations at S60 and T66, i.e., S60D and S60E, promotes mitophagy, while one carrying mutation impeding phosphorylation, i.e., S60A or S60/T66A, inhibits mitophagy. Interestingly, we also found that dephosphorylation of S60 and T66 triggers ubiquitination and degradation of BNIP3. We also identified MAPK8/9 and PP1/2A as the kinase and phosphatase modifying BNIP3 S60 and T66, respectively, using specific inhibitors and siRNAs. Further, we provided evidence through overexpression and knockdown experiments that phosphorylation of BNIP3 at S60 and T66 by MAPK8/9 promotes mitophagy, whereas dephosphorylation of BNIP3 at the same residues by PP1/2A leads to its ubiquitination and degradation, thereby inhibiting mitophagy. Finally, by detecting the expression and activity of MAPK8/9 and PP1/2A under hypoxia conditions, we observed that MAPK8/9 was transiently activated by hypoxia and inactivated after prolonged hypoxia, while PP1/PP2A expression did not change significantly under the same conditions. Collectively, we revealed that MAPK8/9 phosphorylates BNIP3 at S60 and T66, and phosphorylated BNIP3 interacts with MAP1LC3 to promote mitophagy in the early stages of hypoxia (Fig. 1). Subsequently, persistent hypoxia leads to MAPK8/9 inactivation, which results in S60 and T66 dephosphorylation by PP1/PP2A and BNIP3 degradation via the ubiquitin-proteasome pathway, thereby inhibiting mitophagy during prolonged hypoxia (Fig. 1).

**Figure 1.**
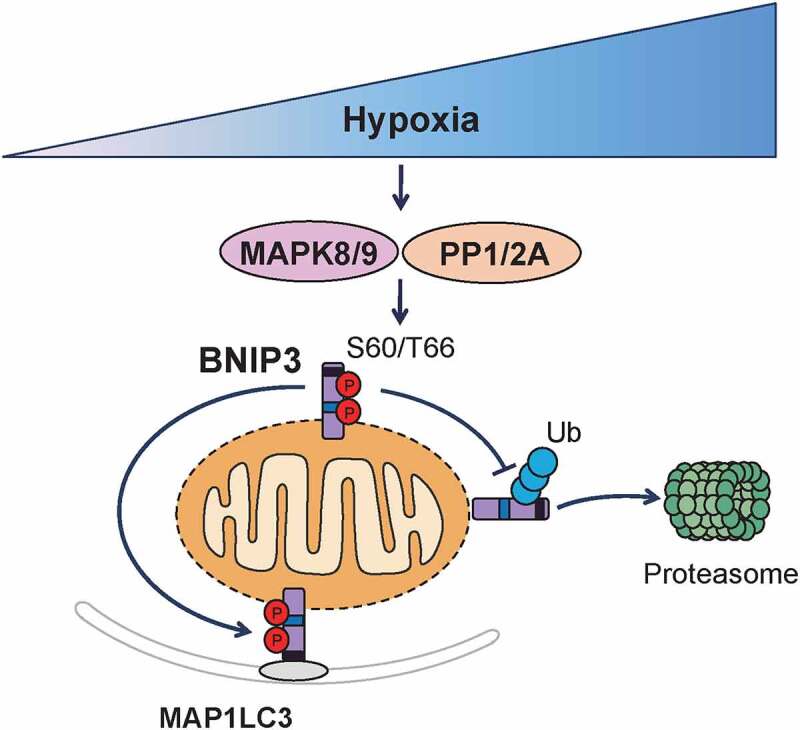
Phosphorylation and ubiquitination of BNIP3 regulate mitophagy under hypoxia conditions. BNIP3 is phosphorylated at S60 and T66 by MAPK8/9, and phosphorylated BNIP3 interacts with MAP1LC3, leading to an induction of mitophagy in the early stages of hypoxia. Upon prolonged hypoxia, MAPK8/9 is inactivated and BNIP3 is dephosphorylated by PP1/PP2A. Dephosphorylated BNIP3 is ubiquitinated and degraded via ubiquitin-proteasome pathway, leading to an inhibition of mitophagy.

In addition to transcriptional regulation, our recent study revealed a post-translational modification pathway to regulate BNIP3-mediated mitophagy under hypoxia by also controlling the levels of this mitophagy receptor. Several recent studies have focused on the role of phosphorylation in BNIP3-mediated mitophagy and reported additional kinases and phosphorylation sites. However, the mechanism of BNIP3 ubiquitination and its role in mitophagy is far to be understood. Moreover, the precise regulatory mechanism between phosphorylation and ubiquitination of BNIP3 that we have unveiled remains to be elucidated. Finally, whether there is a crosstalk between the BNIP3 phosphorylation mediated by different kinases, and whether BNIP3 phosphorylation is involved in other pathophysiological processes is unknown. These are some of the aspects of BNIP3-mediated mitophagy that future studies will need to address.
